# Barriers and enablers for the development and implementation of allied health clinical practice guidelines in South African primary healthcare settings: a qualitative study

**DOI:** 10.1186/s12961-017-0243-3

**Published:** 2017-09-15

**Authors:** J. M. Dizon, K. Grimmer, Q. Louw, S. Machingaidze, H. Parker, H. Pillen

**Affiliations:** 1 International Centre for Allied Health Evidence (iCAHE), University of South Australia, City East Campus, North Terrace, Adelaide, 5000 Australia; 2 Centre for Evidence-Based Health Care (CEBHC), Faculty of Medicine and Health Sciences, Stellenbosch University, Francie van Zijl Drive, Tygerberg, 8000 Cape Town, South Africa; 3 Department of Physiotherapy, Faculty of Medicine and Health Sciences, Stellenbosch University, Francie van Zijl Drive, Tygerberg, 8000 Cape Town, South Africa; 4 Cochrane South Africa, South African Medical Research Council, Francie van Zijl Drive, Parow Valley, 7505 Cape Town, South Africa; 5 European and Developing Countries Clinical Trial Partnership (EDCTP), Francie van Zijl Drive, Parow Valley, 7505 Cape Town, South Africa

**Keywords:** Allied health, Clinical practice guidelines, Barriers, Enablers, Implementation, Local contexts, South Africa

## Abstract

**Background:**

The South African allied health (AH) primary healthcare (PHC) workforce is challenged with the complex rehabilitation needs of escalating patient numbers. The application of evidence-based care using clinical practice guidelines (CPGs) is one way to make efficient and effective use of resources. Although CPGs are common for AH in high-income countries, there is limited understanding of how to do this in low- to middle-income countries. This paper describes barriers and enablers for AH CPG uptake in South African PHC.

**Methods:**

Semi-structured individual interviews were undertaken with 25 South African AH managers, policymakers, clinicians and academics to explore perspectives on CPGs. Interviews were conducted by researcher dyads, one being familiar with South African AH PHC practice and the other with CPG expertise. Rigour and transparency of data collection was ensured. Interview transcripts were analysed by structuring content into codes, categories and themes. Exemplar quotations were extracted to support themes.

**Results:**

CPGs were generally perceived to be relevant to assist AH providers to address the challenges of consistently providing evidence-based care in South African PHC settings. CPGs were considered to be tools for managing clinical, social and economic complexities of AH PHC practice, particularly if CPG recommendations were contextusalised. CPG uptake was one way to deal with increasing pressures to make efficient use of scarce financial resources, and to demonstrate professional legitimacy. Themes comprised organisational infrastructures and capacities for CPG uptake, interactions between AH actors and interaction with broader political structures, the nature of AH evidence in CPGs, and effectively implementing CPGs into practice.

**Conclusion:**

CPGs contextualised to local circumstances offer South African PHC AH services with an efficient vehicle for putting evidence into practice. There are challenges to doing this, related to local barriers such as geography, AH training, workforce availability, scarce resources, an escalating number of patients requiring complex rehabilitation, and local knowledge. Concerted attempts to implement locally relevant CPGs for AH primary care in South Africa are required to improve widespread commitment to evidence-based care, as well as to plan efficient and effective service delivery models.

## Background

The use of clinical practice guidelines (CPGs) to facilitate implementation of evidence-based practice (EBP) has been the subject of research in higher income countries over the past three decades [1–4]. Interest in CPGs has been underpinned by a desire to provide high quality healthcare based on the best available scientific evidence, and reduce inappropriate variations in practice [5]. CPGs have been proposed as an effective and practical vehicle by which to address these concerns [5]. Development of high quality CPGs includes systematically searching, appraising and collating available scientific evidence, and incorporating the views of end-users into documents that can guide local practice [1–5].

Despite the promises of CPGs, there is inconsistent evidence internationally for their effectiveness in improving resource utilisation or patient outcomes [6–8]. These surprising results may partly be explained by the breakdown of processes related to implementation, uptake or use of CPGs. Across health disciplines (medicine, nursing, allied health (AH), dentistry) there are generally positive attitudes to using CPGs as a way of putting evidence in practice. Regardless of a country’s geographic location or its economic status, health system pressures almost universally demand the rapid uptake of EBP by policymakers and healthcare providers to ensure that scant resources are wisely allocated, and treatment optimises patient and economic outcomes for those living with morbidity. Despite discipline differences, competencies and areas of practice, similar reasons are proposed for not actually doing so. Commonly reported barriers are lack of time, lack of ready access to CPGs, lack of understanding about CPGs and how to evaluate their quality, disagreement with CPG recommendations, unwillingness to change practices, peer-pressure, lack of managerial and organisational support, and differences between the research recommendations and clinical realities [5, 9–15].

Within low- and middle-income countries (LMICs), the development and implementation of CPGs can be problematic for health systems experiencing the often dramatic effects of demographic and epidemiological transitions. This is because local evidence of effective care for local conditions may be scant, and application of evidence from other countries may be inappropriate to local contexts (local epidemiology of disease, different patient needs, variable local resources, constraints on local healthcare budgets). The healthcare focus in LMICs such as South Africa is moving rapidly from containing and managing high mortality from communicable diseases, to the management of a previously unforeseen tsunami of morbidity attributed to living with chronic communicable diseases and the sequelae of non-communicable disease [16]. There is hence an increasing focus on rehabilitation and management of disability in South Africa for which there is scant locally relevant research evidence [17–20]. Within South Africa, AH therapies play significant roles in minimising functional loss resulting from chronic managed illness (e.g. HIV and tuberculosis), lifestyle-related chronic diseases (e.g. diabetes, hypertension, respiratory, circulatory), and neurological disease or trauma sequelae (e.g. birth defects, neurological or orthopaedic trauma or disease) [21].

This paper presents findings from a recent study of South African AH primary healthcare (PHC) voices, regarding PHC CPG activities. This was undertaken as part of Project SAGE, a South African Medical Research Council Flagship project 2014–2017 [22]. The PHC definition used to frame this research was that of the National Department of Health South Africa [23].

PHC is centred on the individual, the family and the community. The support they receive for treating and preventing disease, and for protecting, maintaining and improving their health, is integrated across health and health-related sectors. These include housing, water, sanitation, agriculture, education, social welfare, environment, trade and commerce, etc. Within the health system, the health services provide the principal and most direct support to the community.

The pressure on South African AH PHC services to anticipate and address community rehabilitation needs is immense, but there are also significant external pressures on the AH collective to justify growth in the AH workforce and the importance of ongoing training as well as increasing access to resources to meet patient demand. This is because of the continuing national prioritisation of medical services to deal with ongoing disease burden and a scarce national health budget that cannot stretch to cover the growing need for rehabilitation. Thus, whilst there is the will to adopt CPGs, this will is often undermined by health resource allocation, budgetary constraints and health policy priorities that impact on the feasibility and practicality of implementing CPGs in AH PHC.

LMICs usually have few resources to develop and implement locally written CPGs, or even to conduct primary research to explore local context issues. To address this gap, they often adopt CPGs from other countries. However, in many instances, these CPGs come from high-income countries (HICs) with different health systems, workforce training and workforce availability, and thus the guidance in CPGs may not be appropriate for implementation in local South African contexts [17, 18]. Consequently, research underpinning HIC CPGs is of potentially limited value for AH in LMICs [24–26].

The term ‘AH’ is used collectively in our research, referring to non-nursing and non-medical practitioners who provide rehabilitation services in PHC in South Africa, and who operate within conventional boundaries of biomedicine and EBP. However, an international issue relevant to rehabilitation across HICs and LMICs is the lack of an agreed definition of who belongs in the AH collective [27]. There is unresolved and often counter-productive debate about overlap of AH roles, ‘ownership’ of patients (and parts of patients) during rehabilitation, and lack of clear understanding within or outside AH disciplines, of what each AH discipline contributes to rehabilitation outcomes [28, 29]. Thus, in our research, we needed to hear a broad range of views about AH engagement in rehabilitation in South African PHC. Thus, we established a broad reference sample for this study, as there were few individuals, or organisations, which could provide a comprehensive overview of what AH did in this space [30]. Taking a transparent and defensible approach to sampling for investigative questions such as ours, when there are poorly defined reference samples, is supported by qualitative sampling theorists [31, 32].

Prior to undertaking this research, we established the types, content and quality of freely available South African PHC CPGs [33, 34] in order to understand the variability within the current CPGs available to PHC providers. Sixteen CPGs were identified for common South African PHC conditions (diabetes, heart disease, HIV/AIDs, tuberculosis, malaria, maternal and child health, asthma, chronic obstructive pulmonary disease). In HICs, these conditions would generally be managed by multidisciplinary PHC teams and there would be an expectation that multidisciplinary recommendations would be available in CPGs. Yet, none of these CPGs were multidisciplinary and only two of the 16 CPGs contained any recommendation pertaining to AH (both for physiotherapy in asthma and chronic obstructive pulmonary disease) and we found no AH-specific South African CPGs for any condition in our search. The lack of AH-specific CPGs was later confirmed in our interviews.

This paper attempts to describe the multiple factors that may contribute to, or hinder, the development, uptake and use of CPGs by AH policymakers, managers, academics and clinicians in South African PHC.

## Methods

### Ethics

Ethical approval was obtained from the South African Medical Research Council Human Research Ethics Committee (EC002-2/2014), Stellenbosch University Health Research Committee (N14/02/008), and the University of South Australia Human Research Ethics Committee (0000034923). Informed consent was obtained from study participants after receiving written and verbal information regarding the study and participation requirements.

### Design

We used a qualitative descriptive design enacted with individual interviews to report on the barriers and enablers for the development, uptake and use of CPGs in AH practice [31, 32, 35–38]. Qualitative interviews are an appropriate method of investigating how environments interact with particular phenomena [32, 36, 39], which in this case is the South African PHC context surrounding implementation of CPGs in the face of immense workforce pressures and having to deal with increasing patient volume, need and complexity.

### Establishing the sampling

We previously published the sampling frame developed for this study [30]. We sought the perspectives of a range of AH stakeholders involved in the development, implementation or use of CPGs in South African PHC settings. The sample included purposively identified individuals who were actively engaged in AH clinical rehabilitation and/or were involved in activities related to CPGs in clinical practice, research, clinical education, professional development and/or policymaking. The AH disciplines included in this research were physiotherapy, occupational therapy, speech pathology, dietetics/nutrition and podiatry, as these were traditionally engaged in chronic disease and disability rehabilitation in South African PHC settings [30]. Within each AH stakeholder group, potential participants were identified by key informants, or from websites or from telephoning AH professional associations, or by contacting rehabilitation and disability organisations. We employed a method of maximum variation sampling strategy that identified individuals across and within stakeholder groupings. Respondents identified within each stakeholder group were asked to nominate at least one other person from that same group to facilitate within-group snowball sampling [30, 36, 37].

### Data collection methods

Semi-structured interviews were used to elicit individual perspectives on barriers and enablers to writing and implementation of CPGs in PHC rehabilitation for chronic disease and disability. All interviews were conducted in English within the respondent’s workplace in a quiet and confidential environment. English was the common language of all participants, many of whom spoke multiple languages.

Five researchers (KG, QL, SM, JD, HPa) conducted the interviews in pairs, with each dyad featuring a South African health researcher (QL, SM, HPa) and two researchers with experience in CPG writing and implementation (KG, JD). This approach facilitated in-depth exploration within the interviews of factors related to local context as well as technical matters. All interviewers had received training in qualitative methods and interviewing techniques prior to commencement of the study.

The interview schedule allowed exploration of a broad range of issues related to CPG need, writing and implementation in PHC settings in South Africa. The interview questions sought to obtain information on existing frameworks, supports and activities that are used to develop and implement CPGs, current roles, skills and availability of resources available to support implementation of CPGs and what additional resources are required, barriers and facilitators for the development and implementation of CPGs, and the contexts in which CPGs are formulated and implemented. The interviewer also encouraged exploration of responses using a combination of conventional interview techniques (e.g. probing questions, seeking clarification, confirming answers if required and presenting reflections) [35, 38].

Interviews were conducted from May to September 2015. Each interview lasted for approximately 1 hour. Where consent was provided by respondents, the interview was recorded using an electronic audio recorder. Where informants did not consent to the audio recording of interviews, written notes regarding content, tone and body language were recorded by the interviewer. Recruitment to interviews, through snowball sampling, continued until consensus agreement was reached regarding data saturation for each stakeholder group. The research team predetermined that data saturation would be claimed if no new codes had emerged in at least two consecutive interviews.

### Data analysis tools

The qualitative data management software Atlas.ti (http://atlasti.com/) was used for the first analysis exercise, and data management software *NVivo Pro* Version 11 was used for the second analysis exercise (http://www.qsrinternational.com/nvivo-product/nvivo11-for-windows/pro).

### Interpreting the data

Content analysis was used as a strategy for interpreting transcribed interviews and the field notes, which is a preferred method of analysis when using a qualitative descriptive design [35–37]. Analysis was undertaken twice using different approaches.

- Firstly, a coding frame was established by HPa, QL and JD. Family codes, themes and subthemes were identified. Once this step had been completed, the three respective bundles of interviews were combined in *Atlas.ti*, and stored as a single document. The combined document was then run through the *Atlas.ti* system again to extract the respective quotes for each sub-code for further analysis. A large number of categories, themes and quotations were generated using this approach. There was so much complexity and overlap in the results from this process, that it was difficult to classify and interpret the findings clearly.
- A second independent analysis was taken several months later on the same data by KG, JD and HPi, again establishing codes by hand, and then building a second set of categories, themes and quotations using NVivo software. To facilitate this process, a second coding frame was independently developed by two researchers (KG, HPi) who assigned codes to the same three transcripts, with consensus agreement reached regarding suitable codes. Codes were then assigned to the remainder of the transcripts using the analytical tool of constant comparison, with revision of the coding scheme throughout the analytical process [37–39]. Codes were used to identify manifest content within the interviews, focusing on ‘facts’ as perceived by the respondents rather than the meaning underlying these ‘facts’ [37–39]. These codes were then assembled into categories, representing the abstracted manifest content of related codes. Finally, categories were subsumed under themes that formed units of shared latent meaning, as interpreted by the same two researchers (KG, HP).

The results of the two analytical activities were recombined and discussed, to produce the final results and interpretations.

### Rigour

Rigour of data collection and analysis was ensured throughout the study and is described in terms of credibility, transferability and dependability [35–39].

- Credibility refers to the trustworthiness of the data. During the interviews, we repeated the respondents’ answers and asked them to confirm or negate and correct us if necessary. After the interviews were transcribed, we provided a copy to the respondents to confirm the accuracy, and to provide an opportunity to modify or suggest additional content. All sources of data (interview transcripts, analytical memos and field notes noting the content, tone and body language of the respondents) were also triangulated during the analysis to arrive at the codes and themes.
- Transferability refers to the degree to which data can be transferred or generalised from different contexts. To do this, we developed and published a sampling framework using a maximum variation sampling to identify all key players with rich information to share about CPGs in South Africa in order to capture a comprehensive context setting.
- Dependability refers to the stability of data over time. To ensure stability of data, we were transparent with our methods, we undertook constant comparisons of data interpretation and merged two sets of analysis.

## Results

### The sample

As outlined in an earlier publication on how we established the sampling framework [30], we heard from as many different voices as possible. In-depth interviews were conducted with 25 AH stakeholders, and written notes (without recordings) were captured from interviews with four participants. Almost all interviewees wore more than one ‘hat’. They were able to bring a wide range of views from a range of professional activities and experiences, reflecting policymakers at national and four South African provincial levels, managers and/or AH clinicians from all included therapy disciplines from four South African provinces who had knowledge of public and private healthcare settings, representatives of professional associations, two private health insurers, and academics from three tertiary institutions. Data saturation occurred by interview 19, and little more information was elicited in the last six interviews. A summary of respondents’ professional roles is provided in Table 1.

**Table 1:** Summary of respondents’ professional roles

Participant	Mechanism	Policymakers	Academic	District/sub-district public care manager	Clinician public	Clinician private	Medical insurer	Professional association	Consultant	Voluntary professional group member
1	Interview	Prim		Other	Prim					Prim
2	Interview	Other								
3	Interview	Prim	Other							
4	Interview	Other								
5	Interview				Prim				Other	Prim
6	Interview	Other								Prim
7	Interview	Other								Prim
8	Interview		Other							
9	Interview			Other	Prim					Prim
10	Interview		Prim			Other		Prim		
11	Interview					Prim		Other		
12	Interview		Other					Other		
13	Interview	Prim	Prim	Other					Prim	Prim
14	Interview	Other								
15	Interview								Other	
16	Interview	Other								
17	Interview			Other	Prim					
20	Interview	Other			Prim					Prim
23	Interview						Other			
24	Interview	Other								
25	Interview	Other								
26	Interview					Prim		Other		Prim
27	Interview							Other		Prim
28	Interview		Other							
29	Interview		Prim					Other		

*Prim* indicates interviewee (self-nominated) primary role, *Other* indicates the other professional roles played by the interviewees

The key themes from two analyses are reported in Table 2, along with overarching titles for each set of themes. Whilst the wording differed, the intent was the same for the key themes identified in the two analysis approaches. Thus, we were comfortable that we had arrived at a clear understanding of the complexities highlighted in these interviews, regarding AH PHC CPGs.

**Table 2:** Comparison of key themes identified in the analysis approaches

Summary title	Analysis 1	Analysis 2
The collective of AH players	Network of health practitioners Job specification/roles	Interaction between AH stakeholders
The current state of play	Support current guideline activities	Organisational infrastructure Capacities for clinical guideline activities
Putting contextually appropriate CPGs into action	Guideline terms/content indicators	Employing evidence in CPGs Implementing CPGs
Being concerned with CPGs	AH challenges, training	AH factors interacting with political structures Establishing context: moving from complexity to order through CPGs

AH allied health, CPGs clinical practice guidelines

### The collective of AH players

This overarching title encompasses the themes from Analysis 1 (Network of health practitioners and Job specification/roles) and from Analysis 2 (Interaction between AH stakeholders). The AH players mentioned in the interviews included clinicians and managers in different sectors, educators, researchers, policymakers and AH personnel working for private insurance companies who had input into healthcare funding decisions. As all these voices were heard in our interviews, we were comfortable that we had captured data from all relevant voices. This section explores AH stakeholders (players) and type of interactions between them, that facilitate (or prevent) successful CPG application relevant to South African healthcare settings. We heard how AH worked variably in the PHC sector in (1) single discipline clusters as in professional associations, university training programmes and private practices; (2) interdisciplinary clusters in Government and public sector (AH policy portfolios at national and provincial government, AH managers across the public sector institutions, clinicians in specific institutions); and (3) formal and informal multi- and interdisciplinary AH rehabilitation and disability linkages across private, public and academic sectors.

Having a dense network of AH colleagues positioned across sectors was considered a powerful enabler of CPG writing and uptake. These formal and informal connections meant greater access to, and sharing of, information and a better understanding of the relevant forums involved in quality improvement activities.

“*So I think other Chief Directors of Health Programs face the same challenges as I do but I feel incredibly blessed because I have resources that they could never even dream of having. Just because I am in the Western Cape and the Western Cape has all these multiple universities but what we do to bridge the gap is that both ourselves and the clinicians often you find we serve on the National Committees as well. So a lot of our clinical colleagues that are part of our provincial CPGs also sit on National Committees as well and often a lot of the work that we do our national colleagues find out about it and it is often adapted to be national positions.*” (R16)

“*The main aim is to look at professional transformation… a lot of the work that we’re doing is looking at how we can collaborate across disciplines to look at changing rehabilitation generally, because we are very dissatisfied and unhappy with the way in which we do our services in South Africa and also in other countries.*” (R23)

“*So there is a group that works together informally, they came together different stakeholders, different professions to put together this information because the information is there but now to give it, to give it life so that people will be able to implement and also give more information than just a protocol of saying, step one do this, step 2 do this etc., you know, it’s more than just that we need to give information that is not only a clinical level as well.*” (R8)

A formal consultation process where AH stakeholders could comment on draft CPGs was also considered useful in supporting meaningful communication between relevant stakeholders, acting to market the incoming CPG, mitigate practitioner resistance to the proposed recommendations, and assist to contextualise the CPGs to take into account resource limitations (physical and human) within practice environments.

“*So I think in terms of implementation what I’ve seen works really well is when people have been part of the process from the policy development side okay from the word go…When you’ve included them in the process of development and you have actually consulted them and they feel like they’ve been part of development or whatever it is that you are doing and I think also on the implementation guidelines when they are implementation guidelines are easy to use and they are readily available and they are easily adaptable for their context and they are not too rigid.*” (R16)

### The current state of play

This overarching title encompasses the themes from Analysis 1 (Support; Current guideline activities) and from Analysis 2 (Organisational infrastructure; Capacities for clinical guideline activities). These themes refer to the organisational processes, structures and resources required to support the development and uptake of CPGs. In particular, having formal structures and forums for guideline development, establishing structures to support reflective practice and building capacity for guideline development were considered to be important enablers of clinical guideline activities.

Forums for AH planning were an important prerequisite for the initiation, uptake and maintenance of CPGs. These forums should be permanent structures, supported by regular meetings and individuals tasked with their administration and coordination.

“*On a national level we have what we call a national forum where we have representatives per province that meets once per annum. But we don’t meet on our own, we also meet with the other five disciplines, the OT, the speech, audio and the medical and social work. Then we have like a joint national meeting. Then there’s also task groups that come from that meeting and one of them was on developing CPGs.*” (R1)

The lack of experienced supervision was likely to influence the consistent provision of good quality services.

“*There is no supervision, the new clinicians report to a generic clinical manager who is either a doctor or a nurse who has no expertise to advise from a clinical point of view. And that is why my opinion in rehab services in the primary health sector is not of the greatest of quality and they should get a really exceptional therapist.*” (R2)

Whilst there were many reports of vibrant, informal networks and structures which produced CPGs for specific contexts, these lacked legitimacy within healthcare organisations.

“*So there were guidelines, but they were set up by a committee called MADAC (which was the mobility assistive devices advisory committee). And it was then discovered that we actually had no legs to stand on because we’re sanctioned by province, so all the decisions made at that level, we’ve been told basically that they’re not worth anything because it wasn’t okayed officially…*” (R6)

In the presence of large and diverse workloads, clinical supervision and/or professional mentoring was considered important in supporting AH clinicians, managers and policymakers to better reflect on current practice and its alignment with EBP. It was apparent that limited opportunities for reflection on current practice meant that little space was left for quality improvement activities, including initiation, writing and uptake of CPGs.

“*…what she* [occupational therapy research student] *found is that some of them have no mentoring and supervision at all, some of them have from more experienced colleagues, but it varies widely across the provinces and across even the placements within a province. So there is no formal structure, it’s very hit or miss, some are not given anything at all and some are given reasonable mentoring.*” (R9)

Inadequate knowledge of the process of clinical guideline writing and implementation, and a deficit of skills required for these processes were considered major barriers to the development of CPGs.

“*It’s lack of knowledge, so clinicians don’t know how to write guidelines, they don’t know how to look at literature and appraise it so that’s been the one thing and the other thing has just been time. So those are the two things that have kind of stopped us from doing it, so it’s capacity issue in the knowledge skill area as well as just the time.*” (R9)

### Being concerned with CPGs

This overarching title brings together themes from Analysis 1 (AH challenges; Training) and Analysis 2 (Establishing context and moving from complexity to order through CPGs; AH factors interacting with political structures). A narrative emerged among AH stakeholders of a dissonance between the perceived needs of users of healthcare and their ability to provide services to fully meet these needs. The PHC system was considered inadequately resourced and infrastructure poor, resulting in perceptions of unmet need amongst healthcare users. This could be observed in the way in which respondents repeatedly shifted between a language describing the frustrating realities of practice and a language reflecting their aspirations and desires for an idealised practice.

“*So, so, ja, there’s, there’s still there are needs, there are needs everywhere*” (R12)

For many respondents, it was a shifting burden of disease, from communicable to non-communicable diseases, that was contributing to an increasing complex environment for delivering AH services in PHC settings. This was partly due to the fragmentation of service delivery as healthcare systems were trying to accommodate both traditional models of service delivery with new services designed to respond to emergent chronic diseases.

“*…one of our family physicians did a study where they found that 80% of the facilities that of the patients coming into our facilities had chronic conditions. Of that 80% more than half had two or more conditions but you still have an HIV clinic, a diabetic clinic…*” (R16)

In this environment of complexity and unmet needs, CPGs were used to rationalise the use of scare resources, driven by the needs of health insurers and limited healthcare budgets. Efficiency was particularly important as funders of services sought to determine what interventions, delivered to what extent, would result in meaningful outcomes for users of healthcare.

“*CPGs interface with quality in terms of national health insurance, but also possibly prescribed minimum benefits, so the insurance industry in terms of what they will remunerate and not remunerate. So my interpretation or my understanding of it is…we need to develop these so that clients will have a best-practice, latest evidence, comprehensive service delivered to them…*” (R18)

Respondents also reported greater professional expectation to justify their treatment decisions within an EBP framework, brought about by a new generation of AH professionals entering the workforce and external legislative and financial health system pressures.

“*…the younger group of* [therapists] *coming out of university now they are drilled like you’re doing evidence base…* [universities] *challenge the students to say, if you say that what is the evidence backing that.*” (R11)

“*I think the new legislative environment in which there is an expectation that you will be able to show the benefit of what you do.*” (R18)

We also heard about the chaotic space in which AH professionals and policymakers competed for influence, and the ability to place CPGs on the policy agenda. Key to respondent narratives was the struggle to locate access to decision-makers within the healthcare system. Within South African PHC contexts, there were variable perspectives regarding who was responsible for CPG writing or implementation. Numerous indications were provided of the complexity of the relationships between stakeholders operating in different sectors, and at national, provincial and district levels. This complexity was compounded by the separation of funding and policy responsibilities between levels of government and attempts to differentiate between clinical and policy issues.

“*You would have imagined that sitting at National we have a direct influence on what happens there but we don’t. Even when you, you develop a policy when you reap the implementations it’s still a negotiated because the budget that Minister of Health, you know, gives to province is controlled at that level so provinces are responsible for identifying priorities and you know deploying those resources in areas which they think are priority. Sometimes they are in line with what National is doing sometimes they’re not and when they are not, you, you have a challenge to prioritise.*” (R12)

This confusion may partly be attributed to the observation that the medical agenda was strong at all levels of decision-making, and political elites tended to exert undue influence over the healthcare agenda, overshadowing the needs and support required to implement contextually relevant AH CPGs.

“*But there are some policies that are more like directive policies that come straight from, I mean from the political statement by the minister of health or the vice president or the president himself, so those they just come as they are and then we have to now develop guidelines, provincial implementation guidelines as a province that will be used by implementers either at a district level, at the institutional level or community level. So that’s how we manage those kinds of things.*” (R5)

Despite this, there remained various opportunities for AH stakeholders to engage with decision-making structures. Interestingly, government policymakers described significant barriers to initiating development of CPGs, whereas professional associations (e.g. respondent 11 below) were able to access decision-making structures more directly without the same need to pass through the organisational hierarchy of the health sector.

“*We have better access and direct access to the minister of health and director general and so on because we don’t need to go through the managerial structures of the department of health, because we’re an association from outside we can interact with the national department on a high level. So we do have that capacity where people like the guys that were sitting around the table today, they have to go through the bureaucracy of this is my manager, this is my next manager if I don’t have any joy then I go there and they keep on getting blocked, you know.*” (R11)

The political use of language and siding with powerful parties was considered one way in which AH stakeholders could influence the decision-making process.

“*Because the Western Cape department is run by doctors, so most of the hospital managers are doctors, the family healthcare district managers are doctors, top management is doctors and I think that there has always just been a challenge where I think the rehab professionals don't realise that maybe it is because of the language we speak that they don't understand us…they're stuck in a rut, they fight a valiant battle, but because they constantly put themselves in opposition, we don't move forward.*” (R2)

### Putting contextually appropriate CPGs into action

This title encompasses the themes from Analysis 1 (Guideline terms; Content Indicators) and Analysis 2 (Employing evidence in CPGs; Implementing CPGs). Up until this point, the term CPGs has been used to refer to documents that assist with clinical decision-making in a general sense. This departure from the orthodox understanding of ‘clinical practice guidelines’ reflects the lack of consensus amongst AH stakeholders about how CPGs are developed and for what purpose.

“*For me* [podiatrist] *it would be that algorithm, the treatment pathways algorithm: if the patient presents with these things, follow guideline 3b. An (evidence-based) algorithm of the treatment pathway, for me, would be significant.*” (R14)

“*It needs to be step by step preferably if … I like algorithms because they start right at the top and they work your way through depending on the various decisions you make along the way and they have to be very importantly be applicable to the particular audience. So something for a primary care nurse is not the same as something for a GP, which is not necessarily the same as something for a specialist. So the audience need to be taken into account and I think that often is not the case.*” (R20)

The following excerpt demonstrates how CPGs were used to direct implementation of government policy, which may or may not make use of available scientific research.

“[Policy is] *what we want to see, what is the vision of government, it’s translated into a policy right, when it comes to a specific problem…Then as an implementation guideline we need to be drafting something that says what and how and who is responsible…With the protocol now you’re specifying, like clinical protocol, we’re specifying that when you see a child, if a child is under 5 years and they come into your facility, the child must be weighed and how. Now we’re talking about the technical part.*” (R3)

One respondent explained that the framing of policy and guideline documents had very difficult politico-legal implications, meaning that CPGs were just as much tools for influence as they were documents to support EBP.

“*…sometimes it’s easier to get the signatures for a guideline than a policy so then sometimes we change the names of things to a guideline then it get a signature easier…because policies seems you can actually hold the department legally accountable…*” (R8)

In the process of influencing others and promoting successful uptake of CPGs, respondents stressed the need to ensure credibility of CPGs. Credibility tended to be articulated in two ways, one being the quality of the scientific research underpinning the guideline and two being the consensus opinion of domestic and international experts. The latter source of evidence tended to dominate, which was explained by perceptions of irreconcilable contextual differences between the South African healthcare system (and society) and that of economically developed nations producing healthcare evidence. Trust (or mistrust) in the evidence base tended to provide a space for expert opinion to dominate.

“*The production of the guideline is vital. It has to be done with significant credibility and the evidence speaks for itself, so no one will doubt what is written in the guidelines*” (R8)

“*If the evidence is hard to come by, suitable, appropriate and relevant, it’s difficult then to necessarily allow yourself to reason, based on the clinical guideline, if you don’t trust the evidence so much.*” (R18)

Within the context of PHC, there were additional considerations regarding the sourcing of appropriate evidence. The first was that evidence should be interpreted across disciplinary boundaries, reflecting the multi-disciplinary nature of service provision in this setting. The second was that PHC practice was broader in scope and more social in perspective than hospital-based acute care, meaning that new forms of evidence (particularly related to preventative health and rehabilitation) would need to be incorporated into CPGs.

“*Nobody walks in to any of our facilities and say I need a doctor or I need to see a Neurologist or… they don't even know what that is. So to have clinical protocols that are specific to particular clinical areas, I don't think helps in primary healthcare, I think in hospitals create order and they help in terms of how they structure and it’s very linear. In the district health system with the complexities that exist in the how problems get presented to us and if you think about more from what the social constructs are that influence health and wellbeing…*” (R2)

An important consideration in implementing CPGs was its contextual relevance to suit local conditions, acknowledging the diversity of healthcare contexts within South Africa. Although in some cases contextualisation was achieved during guideline development, it appeared more common for health services to perform this function in tailoring evidence for their unique operating environment. A key activity of contextualisation was the development of clinical protocols, procedures and other tools to assist with the process of relating guideline content to clinical encounters.

“*Based on the national guidelines we then develop our own provincial guidelines that would not differ from national but we just add but there’s more specifics for us as a province…when it’s developed nationally it becomes too generic because they look at across all the different provinces and as it comes down to our province we then adapt it to our province but it also becomes generic, so that in terms of implementation each district would then say, this is generic but for us to be able to do this, that is where they develop their implementation plan, for this district this is how we’re going to do it.*” (R5)

The effective communication and distribution of CPGs was also considered an important facilitator of uptake. However, approaches to dissemination tended to use less comprehensive approaches such as the use of health service circulars and personal email, with little indication of who had received this correspondence. In some cases, CPGs were made available on intranet and internet forums, although there remained little indication of how these resources were being accessed or used.

“*We have an official system and an unofficial system. The official system is each of those documents gets a Gauteng health circular number and it gets copied and it’s sent to all the service sites and then from there it is supposed to be distributed further down the line…and they then at the registry office of each hospital is supposed to make copies okay but that is more or less not very trustworthy because the registry staff, I don’t know what they do…that system is breaking down along all the links.*” (R10)

The inadequacy of systems for dissemination were offset by attention to the training of AH practitioners and health service managers. Sufficient effort was required to ensure training was delivered in a comprehensive manner and conducted at national, provincial, district and rural service levels. This was particularly true of train-the-trainer formats, which could prevent the flow of information if trainers were not provided with sufficient time and support for the training of others.

“*Look, I think one of the biggest challenges in implementing clinical guidelines, it’s training on the users clinical guidelines. Normally the training…would happen through National doing the training or a service provider doing the training but train a handful of people who are expected to go and train others and then train the trainers kind of approach and that second leg does not always happen so you find people with guidelines that they’re not trained on how to use them and they end up not being used you see.*” (R16)

Evaluation of the implementation process was also seen as a vital activity, particularly when focused on troubleshooting unforeseen issues and modifying guideline content accordingly. Having formal positions or forums within health services dedicated to monitoring and evaluation of guideline implementation facilitated this quality improvement process.

“*Besides the forum meetings, the district co-ordinators would also have their own district and rehabilitation meetings where they would then meet in a particular district, all the hospitals would then be represented and then they meet and that is also where they discuss the challenges they are facing as a district and then are escalated back to us if there are issues that we have to attend to and we get those reports as well to say, this is what is happening pertaining this guideline or this programme and how best we can assist or it’s going smooth, and through the forums as well we get that as well.*” (R5)

## Discussion

To our knowledge, this is the first study that has presented an in-depth exploration and analysis of CPG writing and implementation in AH PHC in a developing country. In this aspect of the research, we believe that we generated new understanding of the issues involved in the writing and uptake of CPGs by AH providers in one LMIC context to support healthcare policymakers, AH managers and clinicians in identifying critical points for intervention, and to identify gaps in knowledge that could be filled by future primary research. The opportunity to undertake this research in South Africa was much valued, given that it was conducted as a corollary to largely medically oriented CPG research [22].

We found that there is a vibrant, enthusiastic collective of AH players who want to be engaged in AH PHC CPGs, but who struggle to find support to do so in terms of funding, recognition, resources and training. Evidence of integrated and valued South African PHC AH interdisciplinary networks contraindicates international reports of AH as an uncoordinated and forced linkage of dissimilar disciplines [40]. Interdisciplinary AH linkages means shared responsibility and shared vision for better quality and more applicable CPGs, service quality improvement and better health outcomes in the currently underfunded and under-recognised PHC areas of rehabilitation and disability in South Africa [41]. Thus, it seems, from our data, that the impact of interdisciplinary South African AH PHC CPG activities could be significantly increased by evidence-informed, targeted lobbying at provincial and national government levels, innovation in identifying where and how better evidence-informed practices are needed, cohesive and informed CPG writing efforts, and targeted and effective evidence-informed implementation of CPGs in areas of need.

Our perspectives on the current state of play of AH CPGs in PHC, and putting contextually appropriate CPGs into action suggests that there is much work to be done, and little time to do it. An important finding was that there is very little consensus regarding who is responsible for developing and implementing CPGs in South African PHC settings, with CPG activities arising from different sectors with limited communication occurring between stakeholders. These individuals or groups do not have the time, energy, funds, capacity or peer support to undertake CPG activities, reflecting similar concerns to CPG development in other settings [2, 10, 14, 34]. Thus, despite of the available literature and guidance on how to develop quality CPGs [42–46], many CPGs in South Africa lack a robust scientific evidence base [33, 34] and are published as protocols, manuals and checklists [33, 34], rather than in a form considered acceptable by international standards.

The tsunami of chronic disease is already obvious in South Africa, and the brunt of managing this is happening in PHC settings. While there is a will to produce locally applicable CPGs that will assist in standardising and improving care, there are also enormous workload pressures of too few AH clinicians on the ground to meet demand, as well as poorly understood clinical complexities associated with treating those patients who are living with chronic (previously fatal) communicable diseases and their sequelae [47]. There are no dedicated, funded CPG writing organisations that can produce best evidence, locally relevant CPGs. This is an area of concern, given the impact that such units have made in other countries (e.g. SIGN, NICE). Moreover, we heard how there are different perspectives in South African AH PHC settings on what constitutes a CPG, and how evidence should be presented to facilitate uptake. This reflects our earlier findings regarding the quality and construction of other South African CPGs for PHC conditions, where the term ‘CPG’ can mean a range of things, and represent variable presentations of evidence [33, 34]. Our interviews also identified significant scepticism regarding the value of non-evidence-based directives and policy, or recommendations that did not have clear underpinning evidence. Writing appropriately contextualised CPGs for South African AH PHC settings requires dedicated training, collegiate and efficient effort in an environment of scarce resources, and confidence to produce something novel and practical. It is essential to take a focused view of the needs of AH stakeholders in PHC settings to ensure appropriate production of relevant and readily applicable local CPGs.

Our research suggests that AH stakeholders across institutions and sectors should be concerned with locally relevant CPGs. Without standard, evidence-based recommendations to assist with assessment, treatment and monitoring, there is the potential for AH care to be wasted (in terms of too much or too little). An understanding of the cost benefits, to both individuals and the country, of effective multidisciplinary AH rehabilitation programmes in PHC settings for patients living with chronic disease would promote the value of AH nationally, and would lead to a value being placed on lives saved.

## Conclusion

There are many challenges ahead in AH PHC CPG activities in South Africa. Although CPG development was seen to be forcibly driven by pressures to make efficient use of scarce financial resources and demonstrate professional legitimacy through EBP, the presence of a supportive organisational infrastructure, supported interactions between AH actors, and being attentive to quality communication and evaluation of CPGs in clinical practice is lacking to facilitate a concerted approach. The ways in which AH stakeholders can produce relevant guidance that enables them to influence standards of care, and interact more effectively with political structures in healthcare systems are areas that warrant immediate attention.
